# Microscopic polyangiitis complicated with ileal involvement detected by double-balloon endoscopy: a case report

**DOI:** 10.1186/1471-230X-13-42

**Published:** 2013-03-02

**Authors:** Masashi Fukushima, Satoko Inoue, Yuichiro Ono, Yoshitaka Tamaki, Hajime Yoshimura, Yukihiro Imai, Tetsuro Inokuma

**Affiliations:** 1 Department of Gastroenterology, Kobe City Medical Center General Hospital, 2-1-1 Minatojimaminamimachi, Chuo-ku, Kobe, Hyogo 650-0047, Japan; 2 Department of Hematology and Clinical Immunology, Kobe City Medical Center General Hospital, 2-1-1 Minatojimaminamimachi, Chuo-ku, Kobe, Hyogo 650-0047, Japan; 3 Department of Neurology, Kobe City Medical Center General Hospital, 2-1-1 Minatojimaminamimachi, Chuo-ku, Kobe, Hyogo 650-0047, Japan; 4 Department of Pathology, Kobe City Medical Center General Hospital, 2-1-1 Minatojimaminamimachi, Chuo-ku, Kobe, Hyogo 50-0047, Japan

**Keywords:** Microscopic polyangiitis, Double-balloon endoscopy, Small intestinal involvement, ANCA-associated vasculitides

## Abstract

**Background:**

Microscopic polyangiitis is characterized by pauci-immune, necrotizing small-vessel vasculitis and an anti-neutrophil cytoplasmic antibody-associated vasculitis. Although gastrointestinal involvement in microscopic polyangiitis is not rare, endoscopic observation of it is extremely rare. To the best of our knowledge, this is the first case report of small intestinal involvement in microscopic polyangiitis detected and followed up by double-balloon endoscopy.

**Case presentation:**

A 70-year-old Japanese woman was transferred to our hospital for close examination of suspected small intestinal lymphoma. Retrograde double-balloon endoscopy revealed various forms of ulcers with redness and edema in the ileum. Histological findings suggested ischemic changes. Because mononeuritis multiplex and a fever spike appeared later, vasculitis was suspected. The perinuclear anti-neutrophil cytoplasmic antibody titer was elevated. Nerve biopsy results suggested vasculitis. From these findings, microscopic polyangiitis was diagnosed. It was suggested that microscopic polyangiitis caused the intestinal involvement. Intravenous pulse cyclophosphamide and oral predonisolone were started. After treatment, perinuclear anti-neutrophil cytoplasmic antibodies decreased to the normal range. Retrograde double-balloon endoscopy after treatment showed ulcer scars and no ulcer.

**Conclusion:**

The cause of gastrointestinal involvement in microscopic polyangiitis is ischemia due to vasculitis. It is difficult to diagnose small-vessel vasculitis by endoscopic biopsy. Although histological evidence of microscopic polyangiitis is important, the treatment should not be delayed by repeating the biopsy, because such delay can result in adverse sequela.

This case report shows that microscopic polyangiitis should be considered as a differential diagnosis when small intestinal changes like those in the present case are observed by endoscopy.

## Background

Microscopic polyangiitis (MPA) is pauci-immune, necrotizing vasculitis of small vessels without necrotizing granuloma. MPA is one of several systemic anti-neutrophil cytoplasmic antibody (ANCA)-associated vasculitides, along with granulomatosis with polyangiitis and allergic granulomatous angiitis. The term MPA was advocated at the Chapel Hill International Consensus Conference in 1994 [1]. MPA involves many organs or systems, including the skin, muscle, lung, kidney, brain, heart, eye, gastrointestinal tract, and peripheral nervous system. According to an analysis of several retrospective European patient cohorts [2], MPA typically affected male patients >50 years of age in most series (female:male ratio of approximately 1:1.5). Among affected organs and systems, kidney involvement is highest (79%–100%), and gastrointestinal involvement occurs in 30% to 50% of patients [2]. Although colonic involvement of MPA observed by endoscopy has been reported [3,4], small intestinal involvement observed by double-balloon endoscopy (DBE) has not been reported so far. We herein report small intestinal involvement of MPA detected and followed up by DBE.

## Case presentation

**Figure 1:**
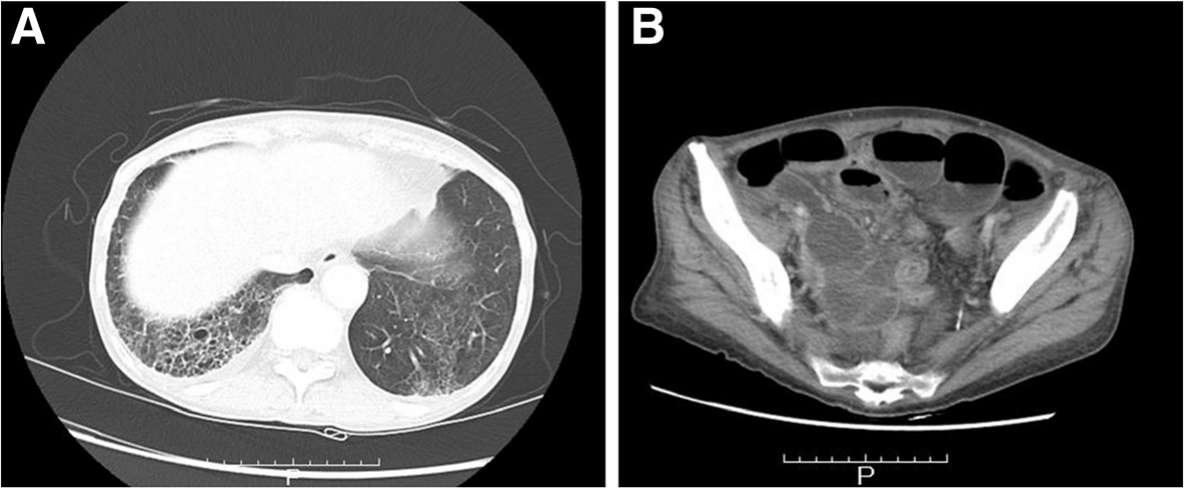
**Computed tomography (CT) findings. A**: Chest CT scan showed honeycomb changes and ground-glass opacities in the lung. These changes suggested interstitial pneumonia. **B**: Abdominal CT scan showed dilatation and wall thickening of the small intestine.

A 70-year-old Japanese woman was admitted to another hospital for paralytic ileus. An abdominal computed tomography (CT) scan showed widespread thickening of the small intestinal wall and ascites. The soluble interleukin-2 receptor level was increased to 2930 U/ml (normal, 220–530 U/ml). She was transferred to our hospital for close examination of the small intestine because intestinal malignant lymphoma was suspected. Her blood pressure and pulse were normal. Her body temperature was 37.4°C. Her weight decreased from 54 to 41 kg in 1.5 years. She had a 2-year history of lower-extremity paresthesia, and lumbar spinal canal stenosis was diagnosed at another hospital. A physical examination revealed signs of anemia. Superficial lymph nodes were not palpable. Livedo reticularis appeared occasionally on the extremities. She was not taking any non-steroidal anti-inflammatory drugs or antibiotics. Peripheral blood analysis showed normocytic normochromic anemia (red blood cell count, 297 × 10^4^/μl [normal, 350–510 × 10^4^/μl], hemoglobin, 8.2 g/dl [normal, 11.1–15.1 g/dl], and hematocrit, 26.0% [normal, 33.5%–45.1%]). Her white blood cell count was 12600/μl (normal, 3900–9800/μl) with 1% eosinophils (normal, 0.0%–8.0%). She demonstrated hypoproteinemia at 6.0 g/dl (normal, 6.5–8.5 g/dl) and hypoalbuminemia at 2.3 g/dl (normal, 3.9–4.9 g/dl). The C-reactive protein level was 6.07 mg/dl (normal, 0.00–0.50 mg/dl). Blood urea nitrogen and creatinine levels were in the normal range. Slight proteinuria (1+) was observed. Hematuria was not observed. Chest and abdominal CT scans showed honeycomb changes and ground-glass opacities in the lung and dilatation and wall thickening of the small intestine (Figure 1).

**Figure 2:**
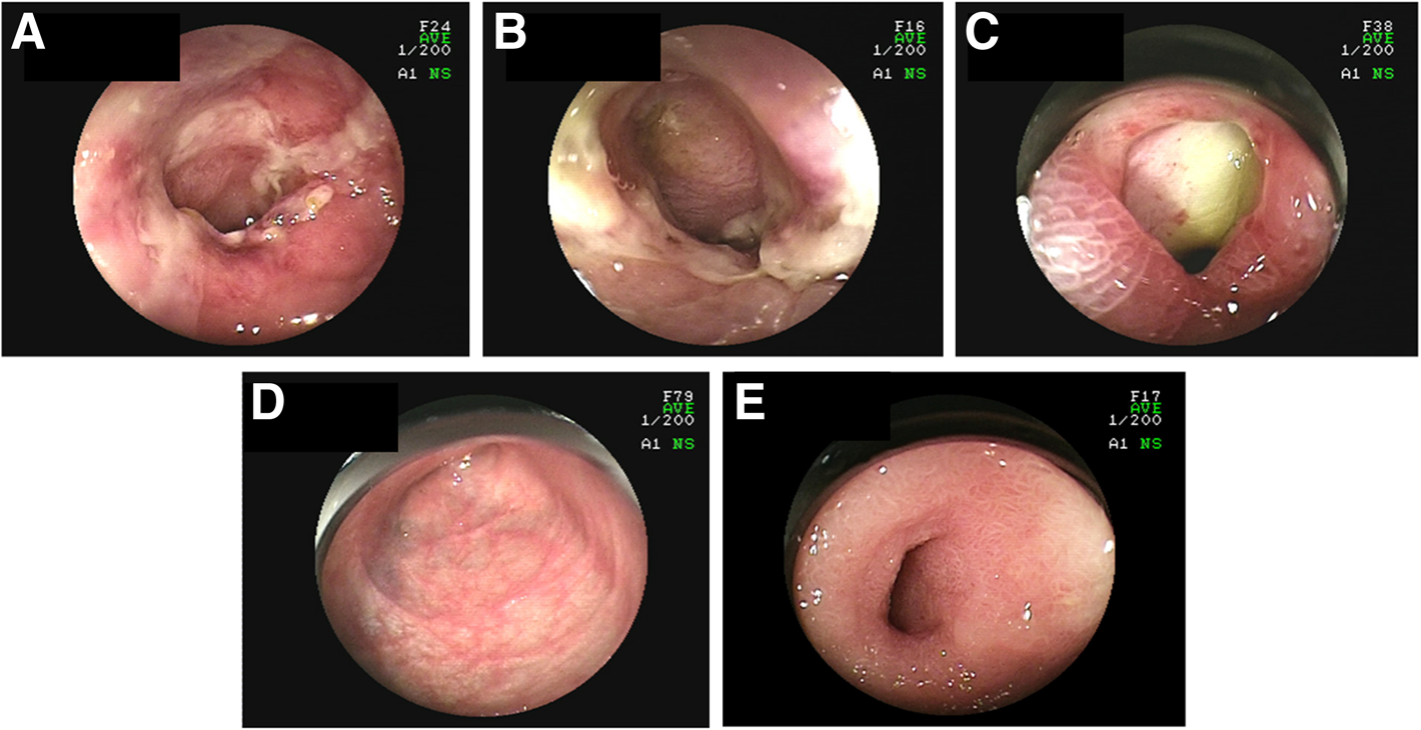
**Endoscopic images of the small intestine before and after treatment. A**: Longitudinal ulcer with redness and edema. **B**: Spiral ulcer. Redness and edema were also observed. **C**: Stenosis. An ulcer was seen beyond the stenosis. **D**: The small intestinal lesions resolved after treatment, and ulcer scars were observed. **E**: The stenosis remained after treatment.

**Figure 3:**
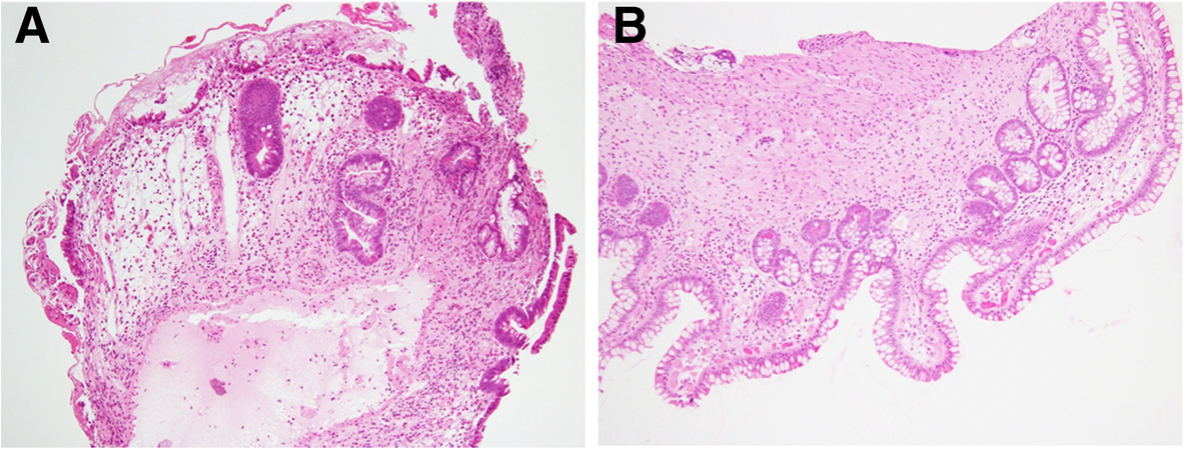
**Pathological findings of the endoscopic biopsy specimens before and after treatment. A**: Before treatment. Microphotograph showed inflammatory cells and crypt destruction and congestion, consistent with ischemic damage (hematoxylin-eosin staining; × 10). Lymphoma cells, granuloma formation, and vasculitis were not observed. **B**: After treatment. Microphotograph showed fibrosis, suggestive of scarring resulting from the previous ulceration (hematoxylin-eosin staining; × 10).

Retrograde DBE showed irregular ulcers in the ileum, which took various forms such as spiral and longitudinal (Figure 2A, B). The most distal ulcer was about 20 cm proximal to the ileocecal valve. Redness and edema were observed around the ulcers, and the borders of the ulcers were unclear. Some of the mucosa between the ulcers was reddish and edematous. A stenosis was also present in the ileum (Figure 2C), and the scope could not pass through the stenosis. No abnormality was detected in the colon. Pathological examination revealed inflammatory cell infiltration (including lymphocytes, neutrophils and eosinophils), intestinal edema and crypt destruction (Figure 3A). Although the interstitial edema and crypt destruction were conspicuous, the inflammatory cell infiltration was relatively mild. These features were suggestive of ischemic changes. It is not known if there was transmural inflammation, because the biopsy specimens did not show the full thickness of the intestinal wall. Lymphoma cells, granuloma formation, and vasculitis were not detected. Retrograde DBE was performed again 1 week later, and the result was similar. Biopsy culture and polymerase chain reaction of tuberculosis were negative. The tuberculin reaction was also negative. The cause of the small intestinal involvement was unknown at that time.

**Figure 4:**
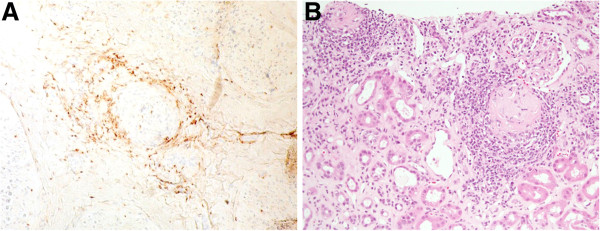
**Pathological findings of the sural nerve and renal biopsy specimens. A**: Many macrophages and T lymphocytes were present around the blood vessels (CD3 antibody staining; × 20). This finding suggested small-vessel vasculitis.(hematoxylin-eosin staining; × 20). **B**: Microphotograph showed glomerulosclerosis in some areas, interstitial fibrosis, and tubular atrophy (hematoxylin-eosin staining; × 20).

Upper-extremity paresthesia and a fever spike appeared several days after the second DBE. Neurological examination revealed sensory and motor disturbances of the extremities. Mononeuritis multiplex, including lower-extremity paresthesia, was diagnosed. Anti-myeloperoxidase ANCA increased to 42.5 U/ml (normal, <9.0 U/ml). Anti-proteinase 3 ANCA, IgG, IgA, IgM, and IgE were within their normal ranges. Sural nerve biopsy was carried out. It did not reveal necrotizing vasculitis; however, it suggested small-vessel vasculitis (Figure 4A). Axonopathy was revealed. Renal biopsy revealed pauci-immune tubulointestinal nephritis (Figure 4B). Esophagogastroduodenoscopy showed no abnormality. Anti-nuclear antibody titers were weakly positive at 1:80. C1q immune complex, lupus anticoagulant, and anti-cardiolipin-β2-glycoprotein I complex antibody were negative. From these findings, the diagnosis of MPA was made. It was suggested that the cause of the intestinal involvement was MPA. Intravenous pulse cyclophosphamide (10 mg/kg, every 2 weeks for the first three treatments and every 3 weeks thereafter) and oral predonisolone (1 mg/kg/day) were started. Six treatment cycles of intravenous pulse cyclophosphamide were performed. Her white blood cell count, C-reactive protein level, and anti-myeloperoxidase ANCA level decreased to their normal ranges. Retrograde DBE about 10 months after the last DBE showed ulcer scars, but ulcers were not detected (Figure 2D). The stenosis was observed in the ileum (Figure 2E); however, she had no gastrointestinal complaints. Pathological examination of the biopsy specimens revealed fibrosis, suggestive of scarring resulting from the previous ulceration (Figure 3B). Paresthesia has remained, but has not deteriorated. Muscle weakness has been recovering gradually.

## Conclusions

Gastrointestinal involvement is common in systemic necrotizing vasculitides, including ANCA-associated vasculitides, polyarteritis nodosa, and rheumatoid arthritis-associated vasculitis [5]. Although gastrointestinal involvement is not rare, it is difficult to diagnose MPA by endoscopic biopsy because it affects small vessels, and endoscopic biopsy specimens are often too small and superficial for a definitive diagnosis. Watts et al. [6] proposed a stepwise algorithm for classification of ANCA-associated vasculitides and polyarteritis nodosa. In this algorithm, in addition to the American College of Rheumatology criteria [7], the Chapel Hill Consensus Conference definitions [1], and the Lanham criteria [8], ANCA and surrogate markers for vasculitis are used because histological data are not available for all patients. Even if there is no histological evidence of necrotizing vasculitis, treatment should be performed when ANCA-associated vasculitides are strongly suspected clinically. Although histological evidence of vasculitis is important, repeat biopsy should be avoided because it can result in delayed initiation of treatment and adverse sequela.

Differential diagnoses of MPA include polyarteritis nodosa, other ANCA-associated vasculitides, collagen disease (such as systemic lupus erythematosus and rheumatoid arthritis), anaphylactoid purpura, and so on. We strongly suspected ANCA-associated vasculitides based on the nerve biopsy findings, clinical symptoms, and blood test results. Signs and symptoms of upper airway involvement, granulomatous formation, asthma, and eosinophilia did not exist. Therefore, MPA was diagnosed with Watts’ algorithm.

In this case, DBE showed multiple irregular ulcerations with redness and edema in the ileum. Ulcer formation was variable. Gastrointestinal involvement of MPA is caused by ischemia due to vasculitis. Endoscopically observed small intestinal involvement in ANCA-associated vasculitides is extremely rare, and only three previous reports of this disorder as observed by endoscopy have been documented [9-11]. Among them, two cases were allergic granulomatous angiitis and one was granulomatosis with polyangiitis. To the best of our knowledge, this is the first case report of MPA complicated with small intestinal involvement detected and followed up by DBE to be documented in the world literature. DBE was an appropriate investigation in this case, because it enabled visualization of the small intestinal lesions. Although it is possible that the ileal lesion could have been observed by colonoscopy, this is by no means certain. It seemed that it would not be possible to reach the stenosis during colonoscopy. The lesions could have been visualized by capsule endoscopy. However, the risk of retention was extremely high because of the stenosis. The patient had severe mononeuritis multiplex and intestinal involvement. We classified her condition as early systemic type, which is one of the categories advocated by the European Vasculitis Study Group [12]. However, her neuropathy was severe, so we started intravenous pulse cyclophosphamide and oral prednisolone. We were able to confirm healing of the intestinal involvement by DBE after treatment. This case report shows that more frequent disorders, such as Crohn’s disease, infections, malignant lymphoma, and drug-induced enteritis, should be suspected first, but that MPA should be considered as a differential diagnosis when small intestinal changes like those observed in the present study are observed by endoscopy.

## Consent

Written informed consent was obtained from the patient for publication of this case report and any accompanying images. A copy of the written consent is available for review by the Series Editor of this journal.

## Abbreviations

MPA: Microscopic polyangiitis; ANCA: Anti-neutrophil cytoplasmic antibody; DBE: Double-balloon endoscopy; CT: Computed tomography

## Competing interests

The authors declare that they have no competing interests.

## Authors’ contributions

MF designed and drafted the manuscript. MF and SI carried out double-balloon endoscopy. SI and TI revised and supervised the manuscript. YO treated the patient. YT and HY carried out neurological examination and nerve biopsy. YI supervised the pathological interpretation. All authors read and approved the final version of the manuscript.

## Pre-publication history

The pre-publication history for this paper can be accessed here:

http://www.biomedcentral.com/1471-230X/13/42/prepub
